# The herbicidal natural product phosphonothrixin is an inhibitor of the riboflavin biosynthetic enzyme L‐3,4‐dihydroxy‐2‐butanone‐4‐phosphate synthase

**DOI:** 10.1002/ps.8791

**Published:** 2025-03-24

**Authors:** Bernd Laber, Yoann Huet, Jörg Freigang, Hansjörg Dietrich, Wolfgang Schulte, David M. Barber

**Affiliations:** ^1^ Research & Development, Division Crop Science Frankfurt am Main Germany; ^2^ Research & Development, Division Crop Science Lyon France; ^3^ Research & Development, Division Crop Science Monheim Germany

**Keywords:** phosphonothrixin, natural product, herbicide, novel mode of action, 3,4‐dihydroxy‐2‐butanone‐4‐phosphate synthase, riboflavin biosynthesis, vitamin B2

## Abstract

**BACKGROUND:**

In our ongoing search for new and environmentally friendly chemical entities that can control weeds *via* new modes of action we reinvestigated the herbicidal potency of the natural phytotoxin phosphonothrixin and researched its mode of action.

**RESULTS:**

Phosphonothrixin displayed broad but non‐selective post‐emergence herbicidal *in vivo* activity in greenhouse tests, albeit at high application rates. Based on a hypothesis on its mechanism of action derived from a literature study we demonstrated that phosphonothrixin significantly increased the thermostability of 3,4‐dihydroxy‐2‐butanone‐4‐phosphate synthase (DHBPS), an enzyme involved in riboflavin (vitamin B2) biosynthesis and inhibited the enzyme competitively with respect to its substrate D‐ribose‐5‐phosphate. Inhibition of DHBPS as the mode of action of phosphonothrixin was confirmed by the elucidation of the X‐ray crystal structure of DHBPS in complex with phosphonothrixin and Mg^2+^‐ions.

**CONCLUSION:**

The natural phytotoxin phosphonothrixin is the first herbicide inhibiting an enzyme involved in vitamin B2 (riboflavin) biosynthesis and represents a prototype of a novel herbicide with a brand‐new mode of action. © 2025 The Author(s). *Pest Management Science* published by John Wiley & Sons Ltd on behalf of Society of Chemical Industry.

## Graphical abstract

An enzyme involved in vitamin B2 biosynthesis, 3,4‐dihydroxy‐2‐butanone‐4‐phosphate synthase (DHBPS), was identified as the target of the natural product phosphonothrixin, representing a novel mode of action for herbicides.

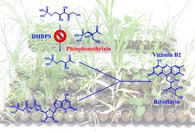

## 1 INTRODUCTION

**Figure 1:**
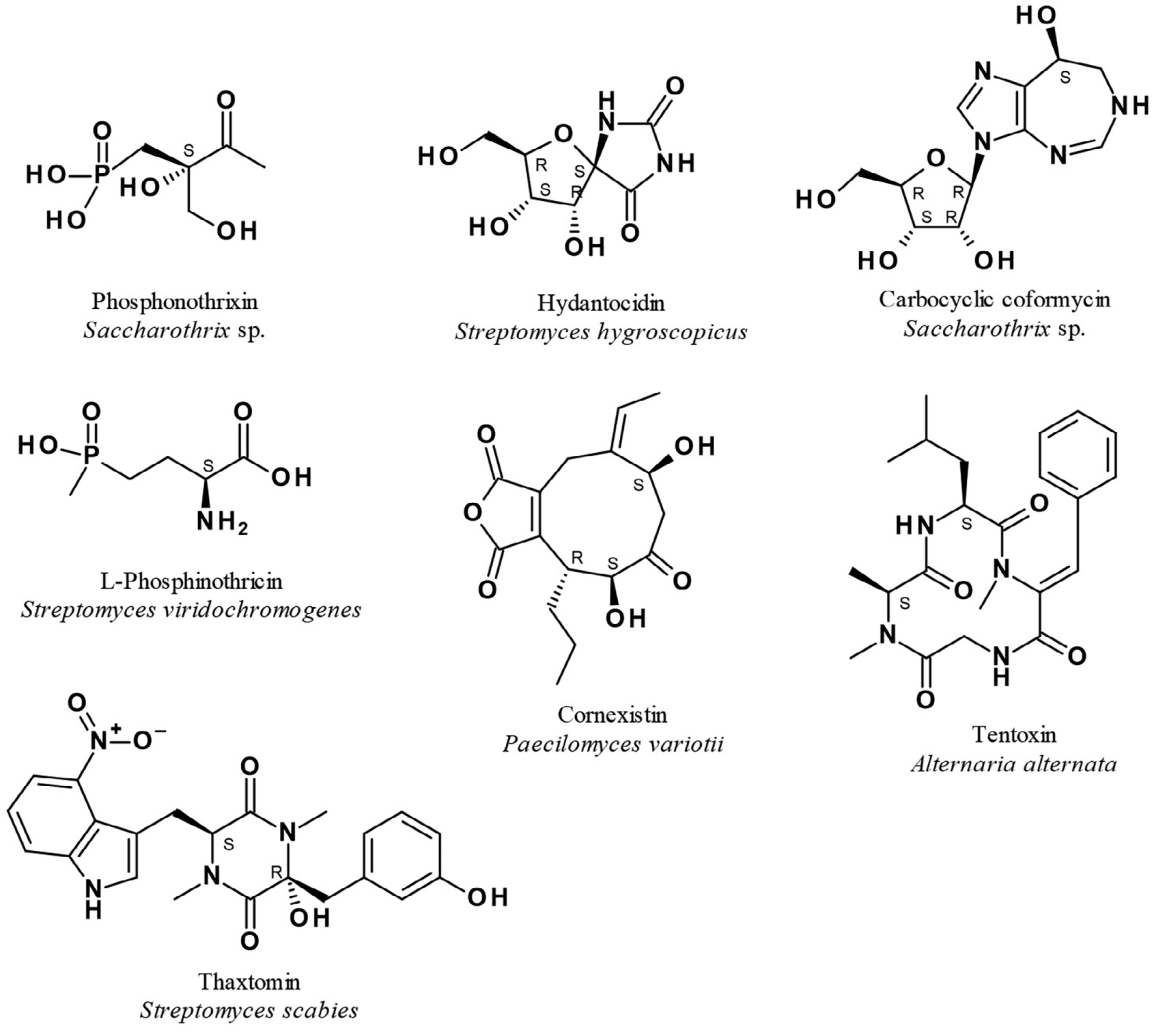
Herbicidal natural products and their sources.

Although herbicides remain the most effective solution for weed control due to their high efficiency and simplicity, they face several challenges, such as more stringent registration procedures and the emergence and growth of resistant weed populations. To provide farmers with new solutions that enable them to fight back against resistant weeds with environmentally friendly herbicides the discovery of chemical entities with new modes of action represents one of the highest priorities of weed control research. Novel herbicides should not only permit effective weed management but should also be environmentally friendly and designed to minimize harm to non‐target species and human health. Due to their structural diversity and evolved biological activity, natural phytotoxins have received considerable attention as potential prototypes of synthetic herbicides. Examples of herbicidal natural compounds are thaxtomin, which disrupts cellulose biosynthesis,^1^ the energy transfer inhibitor tentoxin,^2^ cornexistin, an inhibitor of transketolase,^3^, ^4^, ^5^ hydantocidin as an inhibitor of adenylosuccinate synthetase,^3^, ^6^, ^7^ and the AMP deaminase inhibitor carbocyclic coformycin (Fig. 1).^8^, ^9^ L‐Phosphinothricin, the active principle of the tripeptide bialaphos (L‐phosphinothricyl‐L‐alanyl‐L‐alanine), is a potent inhibitor of glutamine synthetase.^10^ The ammonium salt of racemic phosphinothricin has been commercialized as glufosinate and has reached a market size of about 26 000 metric tons at farm level in 2021.^11^

**Figure 2:**
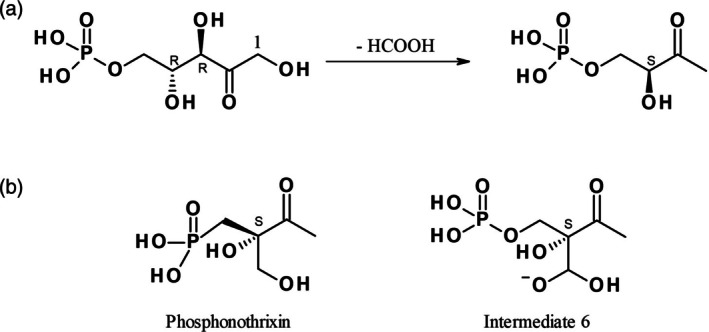
(a) The reaction catalyzed by DHBPS. Carbon atoms 1–3 of the product correspond to carbon atoms 1–3 of the substrate, C‐4 of the product stems from C‐5 of the substrate, C‐4 of the substrate is released as formate together with the H‐atom attached to it, the hydrogen atom at C‐3 of the product is introduced from solvent water.^14^ (b) The similarity of phosphonothrixin (**1**) and the proposed DHBPS reaction intermediate 6.^15^

**Figure 3:**
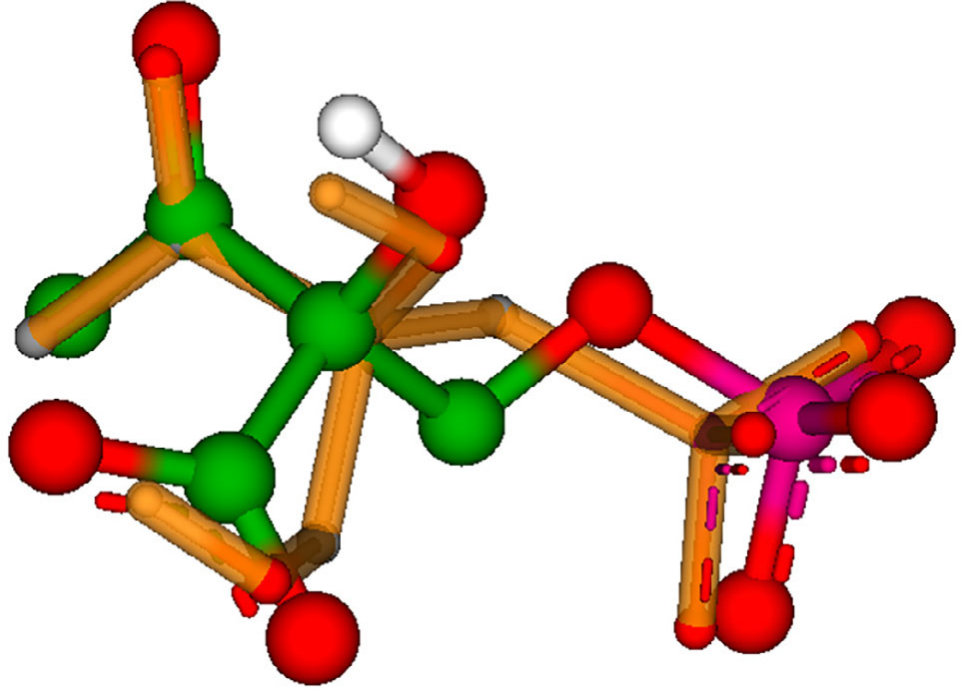
3‐D superposition of *S*‐phosphonothrixin (orange) and intermediate 2 of the catalytic cycle of *Vibrio cholerae* DHBPS^15^ (green), generated with SeeSAR from BioSolveIT.

Phosphonothrixin (2‐hydroxy‐2‐(Hydroxymethyl)‐3‐oxo‐butyl]phosphonic acid) is another natural product that has been reported as an herbicidal compound. It significantly inhibited germination of gramineous and broadleaf weeds and gave rise to chlorosis after foliar application.^12^, ^13^ However, its molecular target has not yet been identified. When studying enzymes of plant specific biosynthetic pathways as putative herbicide targets with a low risk of target‐based toxicological effects, we became interested in the enzymes of the biosynthesis of riboflavin (vitamin B2), the precursor of flavin adenine dinucleotide, an essential cofactor in redox and non‐redox reactions in all forms of life. In one branch of the riboflavin biosynthetic pathway, 3,4‐dihydroxy‐2‐butanone‐4‐phosphate synthase (DHBPS) catalyzes an intramolecular 1,2‐rearrangement reaction of ribulose 5‐phosphate that excises the C4 atom of the substrate as formate to yield L‐3,4‐dihydroxy‐2‐butanone‐4‐phosphate (DHBP; Fig. 2(a)).^14^ Based on the 3D structure of the enzyme, a mechanism for this reaction involving enolization, ketonization, dehydration, skeleton rearrangement, and formate elimination has been proposed^15^ and later been confirmed experimentally.^16^ During an in‐depth analysis of this reaction mechanism, we became aware that one of the proposed intermediates (intermediate 6 in fig. 8^15^) had a striking similarity to phosphonothrixin (**1**) (Figs 2(b) and 3). Based on this observation, we hypothesized that phosphonothrixin (**1**) could be an inhibitor of DHBPS. This prompted us to synthesize phosphonothrixin (**1**) to re‐investigate its herbicidal activity and to assess our hypothesis regarding its mode of action with biochemical assays. In greenhouse trials phosphonothrixin (**1**) showed broad herbicidal activity, albeit at high application rates, covering both grassy and broadleaf weeds. Inhibition of DHBPS by phosphonothrixin (**1**) was confirmed by thermal shift analysis and biochemical activity assays. The target identification was corroborated further by elucidating the X‐ray crystal structures of DHBPS in complex with phosphonothrixin (**1**). These findings make phosphonothrixin (**1**) the first herbicidal natural product to inhibit an enzyme involved in vitamin biosynthesis.

## 2 MATERIALS AND METHODS

### 2.1 General synthesis remarks

General synthesis remarks: All commercially purchased reagents and solvents were used without further purification. All reactions were conducted using magnetic stirring unless otherwise stated. Reactions using temperatures above room temperature (rt) were conducted using a heated oil bath or a Heat‐On block. Yields refer to spectroscopically pure compounds unless otherwise stated. NMR spectra were recorded using a Bruker AVII spectrometer with a Bruker TBI‐probe. The ^1^H and ^13^C NMR shifts are reported in ppm related to the chemical shift of tetramethylsilane. The ^1^H, ^13^C and ^31^P NMR spectra were recorded in a H_2_O buffer solution (0.1 M KCl and 0.1 M NaOH) containing 10% D_2_O that was adjusted to pH 8 using H_3_BO_3_. ^1^H NMR (3.30 ppm) and ^13^C NMR (49.0 ppm) shifts were calibrated using the resonance of methanol as the internal standard. ^31^P NMR shifts were calibrated using H_3_PO_4_ (0.00 ppm) as the external standard.

### 2.2 Synthetic procedures and characterization data of phosphonothrixin (2‐hydroxy‐2‐(hydroxymethyl)‐3‐oxo‐butyl]phosphonic acid, 1)

To a stirred solution of compound **2** (40.0 g, 1.0 eq.) and compound **3** (71.04 g, 0.9 eq.) in CH_2_Cl_2_ (400 mL) at rt was added NaOEt (0.1 eq.) portion wise and the resulting mixture was heated to reflux for 18 h. The reaction mixture was allowed to cool to rt, diluted with H_2_O and extracted with CH_2_Cl_2_. The organic extract was washed with brine, dried over anhydrous Na_2_SO_4_ and concentrated under reduced pressure to afford crude compound **4** (50.0 g), which was used in the next step without further purification. To a stirred mixture of compound **4** (36.0 g, 1.0 eq.) and formaldehyde (37% in H_2_O, 1.5 eq.) at rt was added dimethylamine hydrochloride (1.5 eq.) and the resulting mixture was heated to 65–70 °C for 20 h. The reaction mixture was allowed to cool to rt and then directly concentrated under reduced pressure. The resulting residue was purified *via* column chromatography on silica gel eluting with CH_2_Cl_2_/MeOH to afford compound **5** (10.0 g, 26%). To a stirred solution of 4‐methylmorpholine *N*‐oxide (NMO, 5.85 g, 1.1 eq.) in H_2_O (100 mL) at rt was added OsO_4_ (2.5% in H_2_O, 0.01 eq.). A solution of compound **5** (10.0 g, 1.0 eq.) in acetone (100 mL) was added and the resulting mixture was stirred at rt for 2 h. The reaction mixture was directly concentrated under reduced pressure. The resulting residue was purified *via* flash column chromatography on silica gel eluting with CH_2_Cl_2_/MeOH to afford compound **6** (5.6 g, 49%). To a solution of compound **6** (5.5 g, 1.0 eq.) in CH_2_Cl_2_ (50 mL) at rt was added allyltrimethylsilane (1.2 eq.) and bromotrimethylsilane (3.0 eq.). The resulting mixture was stirred at rt for 2 h. The reaction mixture was concentrated under reduced pressure and the residue was co‐distilled with water to obtain the crude product. The crude product was purified by washing with diethyl ether to afford phosphonothrixin (**1**) (3.9 g, 92%). ^1^H NMR (400 MHz, pH 8 buffer H_2_O + 10% D_2_O): δ 3.80 (d, *J* = 11.8 Hz, 1H), 3.60 (d, *J* = 11.9 Hz, 1H), 2.28 (s, 3H), 2.22 (dd, *J* = 17.9, 15.7 Hz, 1H), 2.04–1.95 (m, 1H). ^13^C NMR (100 MHz, pH 8 H_2_O buffer +10% D_2_O): δ 214.8 (d, *J* = 4.4 Hz), 80.8 (d, *J* = 5.1 Hz), 67.7 (d, *J* = 17.6 Hz), 32.6 (d, *J* = 134.3 Hz), 25.7 (s). ^31^P NMR (162 MHz, pH 8 buffer H_2_O + 10% D_2_O): δ 20.0 (s). MS (ESI, *m/z*): [(M + H)^+^] = 199.1; [(M−H)^−^] = 197.0. The data was in accordance with that previously reported in the literature.^13^, ^17^

### 2.3 Herbicidal greenhouse screening for post‐emergent efficacy

Seeds of mono‐ and dicotyledonous weed plants were sown in plastic pots of 7 cm diameter filled with a clayey silt soil substrate. The plants were maintained for the preparatory phase of germination and initial growth in the greenhouse under optimum growth conditions. Phosphonothrixin (**1**) was prepared for application as an emulsion concentrate and diluted together with 0.2% agrotin (a polyfunctional non‐ionic additive) in water corresponding to a spray volume of approximately 600 L ha^−1^. At a growth stage of 2 to 4 true leaves the test plants were sprayed and then placed in the greenhouse for 3 weeks under optimum growth conditions. Subsequently, the effect of phosphonothrixin (**1**) was assessed visually relative to the untreated controls resulting in herbicidal effects (%) ranging from 0% (no effect = untreated control) to 100% (complete control = died‐off ).

### 2.4 Cloning, expression and purification of DHBPS enzymes

For the expression of the *Arabidopsis thaliana* DHBPS as a C‐terminally his‐tagged fusion protein (see Supporting Information, Fig. S1 for the expressed amino acid sequence) the DHBPS domain (amino acids 112 to 337) of the RIBA1 gene (UniProt ID: P47924) encoding the plastidic bifunctional GTP cyclohydrolase II – 3,4‐dihydroxy‐2‐butanone‐4‐phosphate synthase was first cloned *via* TOPO‐TA cloning into an entry vector and then *via* Gateway Cloning into the pDEST42 vector, which was transformed into the *Escherichia coli* strain BL21(DE3). For enzyme production the bacteria were grown at 37 °C in LB medium containing 100 mg L^−1^ carbenicillin, induced with 1 mM IPTG at OD_600_ = 0.5 and further cultivated at 28 °C. After 16 h the bacteria were harvested by centrifugation. *At*DHBPS was purified with the Quiagen NiNTA Fast Start Kit according to the instructions of the manufacturer. The obtained protein was desalted over GE Healthcare PD‐10 gel filtration columns equilibrated with 50 mM Tris buffer pH 8.0 and stored at −80 °C.

For the expression of the *Zymoseptoria tritici* DHBPS as a N‐terminally his‐tagged fusion protein (see Supporting Information, Fig. S2 for the expressed amino acid sequence) a synthetic gene encoding the RIB3 gene (NCBI accession number SMR48963) optimized for expression in *E. coli* was cloned *via* Nde I/Not I restriction sites into a pET21a vector, which was transformed into the *E. coli* strain BL21(DE3). For enzyme production the bacteria were grown at 37 °C in LB medium containing 100 mg L^−1^ ampicillin, induced with 1 mM IPTG at OD_600_ = 0.5 and further cultivated at 16 °C. After 16 h the bacteria were harvested by centrifugation. *Zt*DHBPS was purified by affinity chromatography on an Äkta Pure device using a 5 mL Ni‐NTA Superflow Cartridges (QIAGEN) equilibrated with buffer A (50 mM Tris pH 8.0, 200 mM NaCl, 10% (v/v) glycerol). The cleared bacteria lysate was loaded at 2 mL min^−1^ and the column was washed with buffer A until reaching a stable UV detection baseline. His‐tagged *Zt*DHBPS was eluted with an imidazole gradient (0–300 mM in buffer A) over three column volumes and the eluate was desalted on a HighPrep 26/10 desalting column (Cytiva) equilibrated in buffer A. A Size Exclusion Chromatography polishing step was performed on a HiLoad Superdex 200 pg preparative SEC column equilibrated in buffer B (20 mM Tris pH 8.0, 100 mM NaCl, 10% (v/v) glycerol). *Zt*DHBPS was recovered in 3 mL elution fractions performed with buffer D at 2 mL min^−1^. The protein was stored at −80 °C.

### 2.5 DHBPS activity assay

DHBPS activity assays were out carried in 96‐well microtiter plates using a modification of the procedure previously described.^18^ The reaction mixture contained 100 mM MES (2‐morpholinoethanesulfonic acid) buffer pH 6.5, 20 mM MgCl_2_, 20 mM D‐ribose‐5‐phosphate (Sigma‐Aldrich R7750), 0.02% Tween‐20, 0.2 mg BSA, 0.5 μg of spinach phosphoriboisomerase (Sigma‐Aldrich P9752) and DHBPS enzyme in a final volume of 110 μL. Assays were initiated by adding enzyme and terminated after 120 min incubation at rt by the addition of 90 μL of a solution of 3.5% α‐naphthol and 0.2% creatine in 2 N NaOH. After an additional 45 min incubation at rt the absorbance was determined at 540 nm. The pI_50_‐value for inhibition by phosphonothrixin (**1**) was determined with the XLFit Excel add‐in version 4.3.1 curve fitting program of ID Business Solutions Limited. For the determination of K_m_, V_max_ and k_cat_ values, DHBPS activity was determined by variation of the D‐ribose‐5‐phosphate concentration in the assay mixture from 0 to 2 mM. Reaction velocities were fitted to the Michaelis–Menten equation with the XLFit curve fitting program.

### 2.6 Thermal stability shift analysis

*At*DHBPS melting curves were determined in 96‐well microtiter plates in a Roche Light Cycler 480. The assay mixture contained 50 mM HEPES pH 7.5, 75 mM NaCl, 2.5 mM MgCl_2_, 7.5x SYPRO Orange and 6.5 μM *At*DHBPS in a total volume of 20 μL. A linear temperature gradient from 25 to 85 °C with a ramping of 3.25 °C min^−1^ was applied. Melting temperatures were determined from plots of the first derivative of the fluorescence intensity *versus* temperature.

### 2.7 X‐ray crystallography

Expression and purification of *Zt*DHBPS were carried out according to the protocols outlined above. Prior to crystallization, the protein was concentrated to 14 mg mL^−1^ in 1 mM MgCl_2_, 100 mM NaCl, 20 mM Tris pH 8.0, supplemented with 1 mM phosphonothrixin (**1**) and incubated for 1 h. The complex was crystallized by sitting drop vapor diffusion at 18 °C using 30% (w/v) polyethylene glycol monomethyl ether 2000 as precipitant. Diffraction data were collected on a D8 Venture diffraction system from Bruker. The structure was solved by molecular replacement with MOLREP^19^ using the structure of DHBPS from *Pyricularia grisea* (PDB‐ID: 1K49) as a search model. Manual model building and refinement were iteratively performed with Coot^20^ and RefMac5.^21^ Data collection and refinement statistics are shown in Supporting Information, Table S1.

### 2.8 *Lemna gibba* growth assays

Growth experiments were performed in sterile 6‐well cell culture plates and were initiated by transferring four *Lemna gibba* fronds per well to 5 mL of K‐medium^22^ pH 4.8 containing 1% (w/v) sucrose and a selected concentration of phosphonothrixin (**1**) and riboflavin determined from previous experiments. The cell culture plates were incubated for 7 days in a growth cabinet at 22 °C and a continuous photon flux density of 75 μE/m^2^s.

## 3 RESULTS AND DISCUSSION

### 3.1 Synthesis of phosphonothrixin (1)

**Scheme 1:**
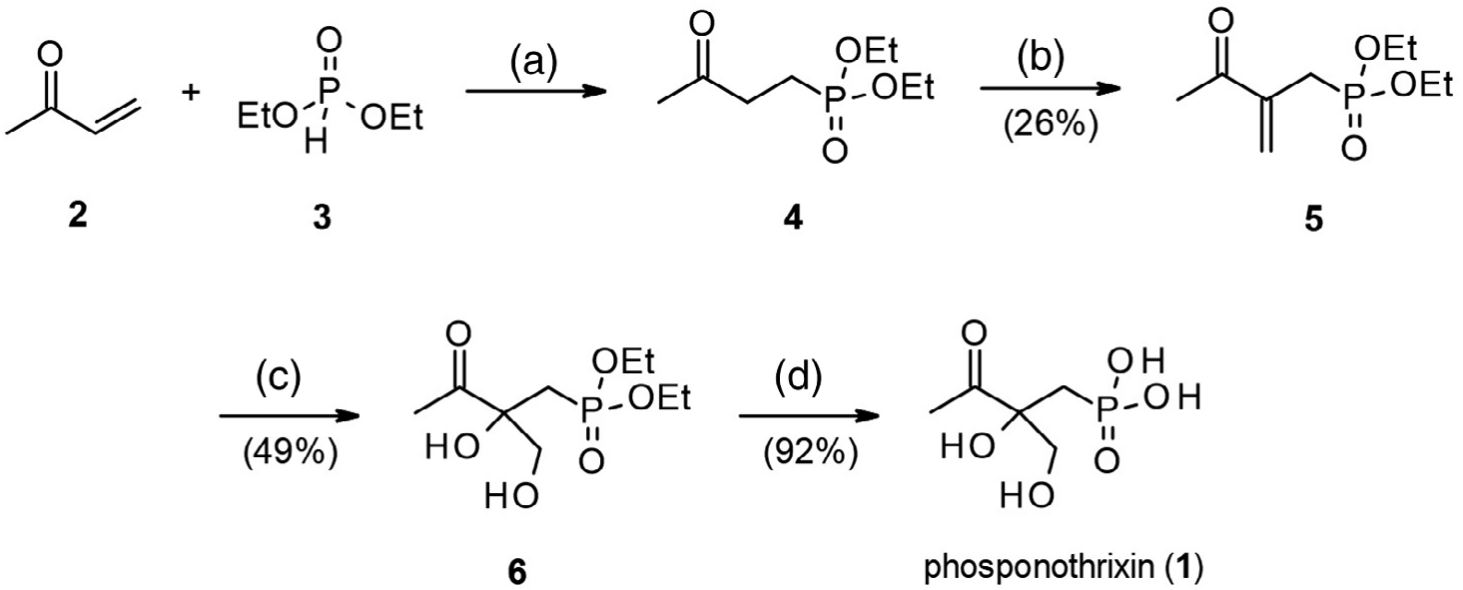
Synthesis of phosphonothrixin (**1**): (a) NaOEt, CH_2_Cl_2_, reflux, used crude; (b) dimethylamine hydrochloride, formaldehyde, 65–70 °C, 26% over two steps; (c) OsO_4_, NMO, acetone, rt, 49%; (d) allyltrimethylsilane, bromotrimethylsilane, CH_2_Cl_2_, rt, 92%.

To access racemic phosphonothrixin (**1**), we employed a synthetic route that started from methyl vinyl ketone (**2**) and phosphonate ester **3** (Scheme 1). Conjugate addition of phosphonate ester **3** to methyl vinyl ketone (**2**) in the presence of sodium ethoxide provided ketone **4**, which subsequently underwent an aldol condensation reaction with formaldehyde to afford alkene **5** in 26% yield over two steps. Dihydroxylation of the alkene using osmium tetroxide and *N*‐methylmorpholine *N*‐oxide (NMO) smoothly yielded di‐alcohol **6**. Lastly, deprotection of the phosphonate ester using a mixture of allyltrimethylsilane and bromotrimethylsilane afforded phosphonothrixin (**1**) in 92% yield.

### 3.2 Herbicidal activity of phosphonothrixin in greenhouse tests

**Figure 4:**
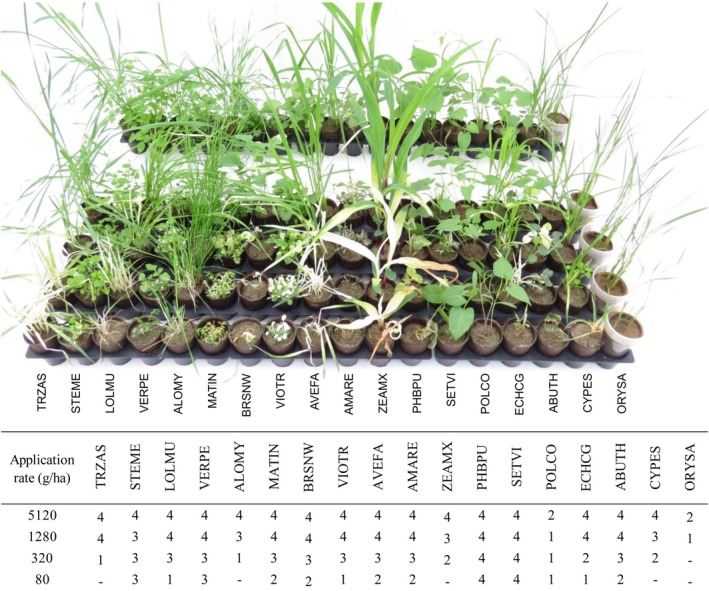
Herbicidal evaluation of phosphonothrixin (**1**) in post‐emergence application. From back to front untreated control (separated) and plants treated with 80, 320, 1280 and 5120 g ha^−1^ phosphonothrixin (**1**), respectively. Rating scale: ‘5’ = > 90% inhibition, ‘4’ = 80–90% inhibition, ‘3’ = 60–79% inhibition, ‘2’ = 40–59% inhibition, ‘1’ = 20–39% inhibition and ‘−’ = < 20% inhibition. Abbreviations: *Abutilon theophrasti* (ABUTH), *Alopecurus myosuroides* (ALOMY), *Amaranthus retroflexus* (AMARE), *Avena fatua* (AVEFA), *Brassica napus* (winter rape, BRSNW), *Cyperus esculentus* (CYPES), *Echinochloa crus‐galli* (ECHCG), *Lolium multiflorum* (LOLMU), *Matricaria inodora* (MATIN), *Oryza sativa* (ORYSA), *Pharbitis purpurea* (PHBPU), *Polygonum convolvulus* (POLCO), *Stellaria media* (STEME), *Setaria viridis* (SETVI), *Triticum aestivum* (spring wheat, TRZAS), *Veronica persica* (VERPE), *Viola tricolor* (VIOTR), *Zea mays* (ZEAMX).

When tested under post‐emergence conditions phosphonothrixin (**1**) showed broad herbicidal activity against 14 agronomically relevant weeds at application rates of 5120 and 1280 g ha^−1^, but lacked crop selectivity (Fig. 4). At application rates of 320 and 80 g ha^−1^ its herbicidal activity was significantly reduced. All affected plants displayed a bleaching phenotype. Even at the highest application rate tested phosphonothrixin (**1**) failed to show any pre‐emergence activity (data not shown). From the weed species that were evaluated, no significant differences were observed between the efficacy obtained when treating monocot weeds and dicot weeds with phosphonothrixin (**1**).

### 3.3 Phosphonothrixin is an inhibitor of DHBPS

In an attempt to validate our hypothesis that phosphonothrixin (**1**) targets DHBPS we first investigated its effect on the thermal stability of *At*DHBPS. When tested at a concentration of 10 μM, phosphonothrixin (**1**) shifted the melting point (deltaT_m_) of *At*DHBPS by 4.0 °C, while any one of 16 different herbicidal compounds tested in comparison induced shifts of the melting point from 0 to 0.7 °C (Supporting Information, Fig. S3). A K_d_‐value of 31.1 ± 2.8 μM and a deltaT_m(max)_ of 22.7 ± 0.6 °C for binding of phosphonothrixin (**1**) to *At*DHBPS was determined from a plot of deltaT_m_ *vs*. phosphonothrixin (**1**) concentration (Supporting Information, Fig. S4). These results indicated a true binding behavior of phosphonothrixin (**1**) toward *At*DHBPS. To investigate this interaction in more detail, we analyzed the *At*DHBPS enzyme kinetically. We determined a K_m_‐value of 150 ± 20 μM for the *At*DHBPS substrate D‐ribulose‐5‐phosphate (Supporting Information, Fig. S5) and identified phosphonothrixin (**1**) as a competitive inhibitor of the enzyme with an IC_50_‐value of 58 ± 11 μM and a K_i_‐value for of 16.6 ± 5.0 μM (Supporting Information, Fig. S6).

### 3.4 X‐ray crystallography

**Figure 5:**
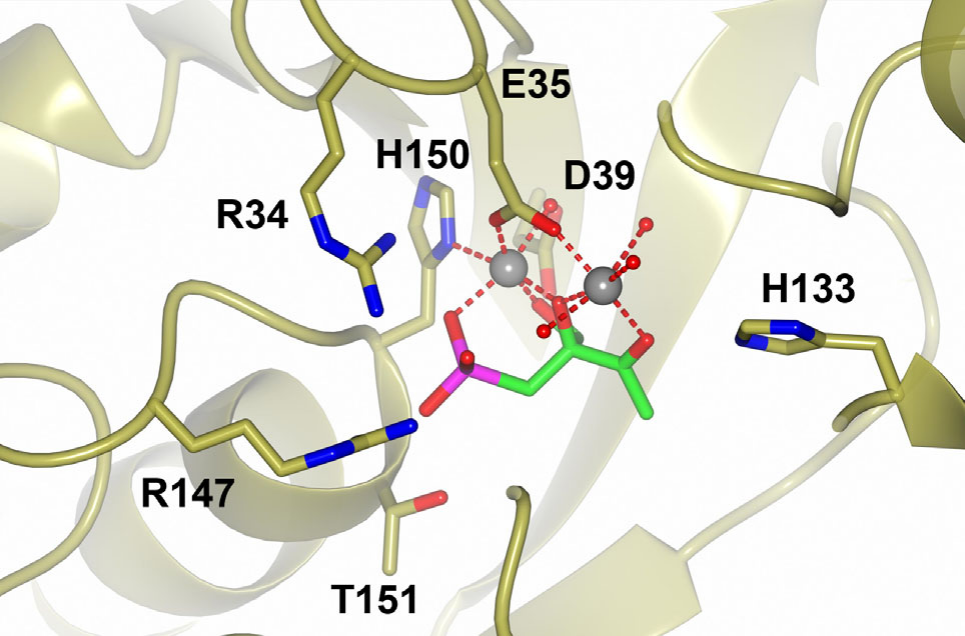
Phosphonothrixin (**1**) bound to the active site of *Zt*DHBPS. Sidechains are only shown for the key residues relevant for inhibitor binding.

To better understand the molecular mechanism of DHBPS inhibition shown by phosphonothrixin (**1**), we tried to determine the X‐ray crystal structure of the *At*DHBPS‐phosphonothrixin complex. Because all our attempts to grow crystals failed, we used DHBPS from the phytopathogen *Zymoseptoria tritici* (*Zt*DHBPS; IC_50_‐value for phosphonothrixin (**1**): 19 μM) as a model system and were able to determine the structure of the ternary complex between phosphonothrixin (**1**), magnesium and *Zt*DHBPS at a resolution of 2.2 Å. In the complex crystal structure (Fig. 5, Supporting Information, Fig. S7), phosphonothrixin (**1**) is tightly bound both by direct interactions with protein atoms and by interactions with the two catalytical magnesium ions in the active site. The phosphono group interacts with the positively charged side chains of R34 and R147, and forms two hydrogen bonds with the main chain nitrogen of H150 and with the sidechain of T151. Additional hydrogen bonds are observed between the carbonyl oxygen of the inhibitor and H133, and between the oxygen of the primary alcohol of the inhibitor and D39. These two and the oxygen atom of the tertiary alcohol serve as ligands for the coordination of the magnesium ions. The coordination sphere furthermore involves a nitrogen atom for the sidechain of H150, two oxygen atoms from the sidechain of E35, an oxygen atom from the phosphono group and four water molecules, resulting in a nearly perfect octahedral coordination of the ions. Taking all of these factors into account, the described interactions nicely explain the affinity of phosphonothrixin (**1**) on a molecular basis.

While the *Zymoseptoria* and *Arabidopsis* proteins share only 45% sequence identity in the DHBPS domain, the inhibitor binding site is highly conserved between the two species. Fifteen out of 17 residues with a distance to inhibitor atoms of less than 5 Å are identical in the two proteins. The two non‐conserved amino acids are N88 and Y92, equivalent to L208 and F212 in *At*DHBPS. While N88 is located close to the inhibitor, it is not directly involved in any interaction with the ligand, and a leucine at this position should not have a direct impact on inhibitor binding. For Y92, a direct hydrophobic interaction between the aromatic ring and the methyl group of phosphonothrixin (**1**) is observed, but a similar interaction would also be expected for a phenylalanine. This high conservation of the binding site implies that the binding mode observed with *Zt*DHBPS is either identical or very similar to *At*DHBPS. This assumption is supported by a superposition of the crystal structure of *Zt*DHBPS and the AlphaFold2^23^ model of *At*DHBPS. The superposition shows that all residues involved in phosphonothrixin (**1**) binding have the same spatial orientation in the model of the *At*DHBPS structure and in the experimentally determined *Zt*DHBPS structure (Supporting Information, Fig. S8).

**Figure 6:**
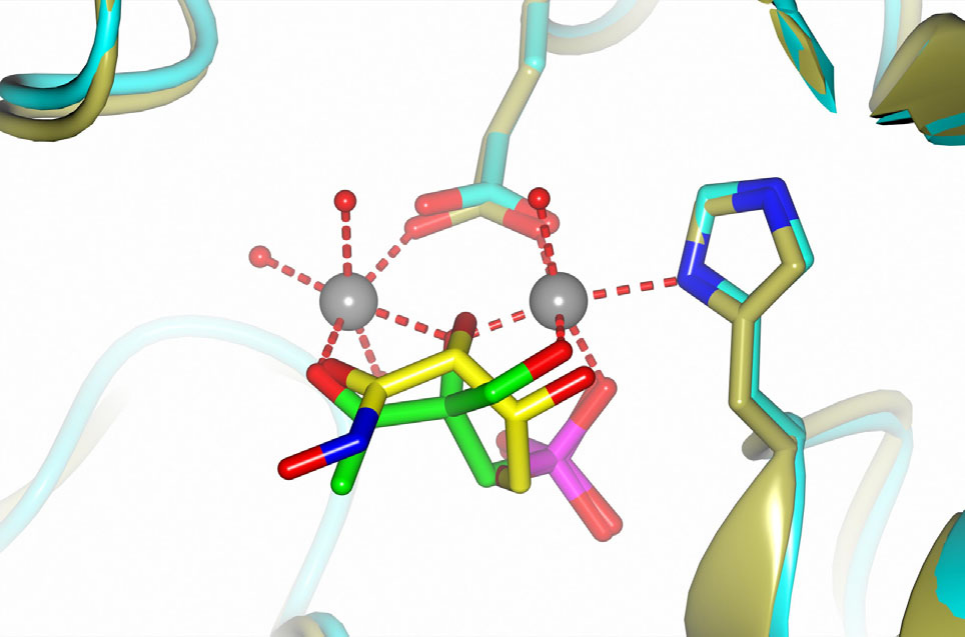
Superposition of phosphonothrixin (**1**) (green) bound to *Zt*DHBS (gold) with 4PEH (yellow) bound to *Vc*DHBPS (cyan). For clarity, only the sidechains that contribute to metal complexation are shown.

The observed binding mode for phosphonothrixin (**1**) to *Zt*DHBPS closely resembles that of the inhibitor 4‐phospho‐D‐erythronohydroxamic acid (4PEH) observed in the crystal structure of its ternary complex with zinc and DHBS from *Vibrio cholera* (*Vc*DHBPS, PDB‐ID: 4P6P).^24^ A superposition of the crystal structures (Fig. 6) shows that the phosphonate of phosphonothrixin (**1**) and the phosphate group of 4PEH are located at equivalent positions, interact with two conserved arginine sidechains and form a hydrogen bond to a main chain nitrogen. In both inhibitor structures, four oxygen atoms are located at corresponding positions to coordinate the two active site ions. It is unexpected that while the oxygen atoms in the inhibitors are connected differently, they can still occupy equivalent positions in the complexation spheres of the ions and form similar hydrogen bonds, resulting in both cases in efficient inhibition.

### 3.5 Effects on *Lemna gibba*

**Figure 7:**
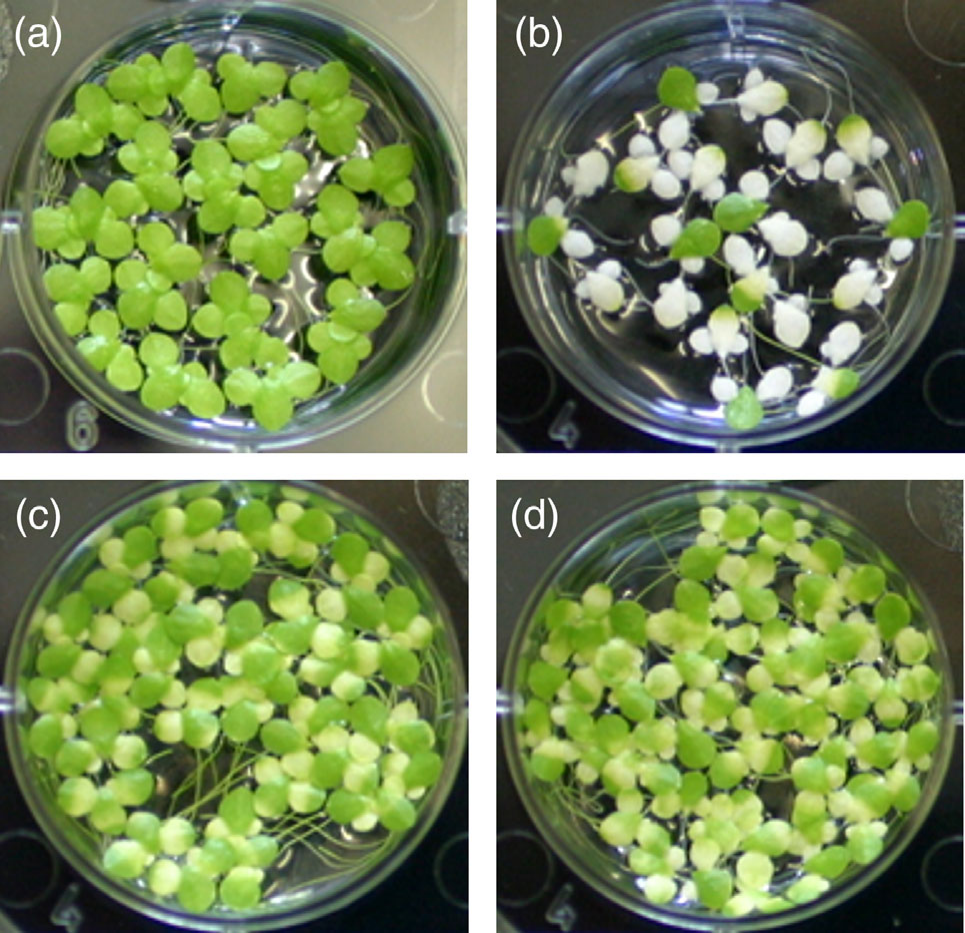
Growth inhibition of *Lemna gibba* by phosphonothrixin **1** and its reversal by riboflavin. (a) Control; (b) 100 μM phosphonothrixin (**1**), (c) 50 μM riboflavin, (D) 100 μM phosphonothrixin (**1**) + 50 μM riboflavin.

Treatment of duckweed plants with 100 μM phosphonothrixin (**1**) led to a significant growth inhibition and induced strong bleaching of the fronds, in accordance with the phenotype observed in greenhouse experiments. Supplementation of the growth medium with the end product of the vitamin B2 biosynthetic pathway, riboflavin, did not affect the growth of the duckweed plants, but for unknown reasons resulted in some mild bleaching of the fronds. However, in the presence of phosphonothrixin (**1**), riboflavin almost completely alleviated the growth inhibition and reversed the bleaching phenotype to the level observed in the experiment where only riboflavin had been applied (Fig. 7). Overall, the results of this experiment can be seen as a strong support for the proposed mechanism of action.

### 3.6 Performance of DHBPS as an herbicide target

Finally, the question of whether phosphonothrixin (**1**), despite its high IC_50_‐value for *At*DHBPS, is strong enough as an inhibitor to produce the observed herbicidal effect should be discussed. Generally, the herbicidal effects of phosphonate herbicides do not correlate with the IC_50_‐values for their target enzymes. For example, for *trans*‐(3‐hydroxy‐3‐(1*H*‐1,2,4‐triazol‐3‐yl)cyclohexyl)‐phosphonic acid (IRL1803), a nanomolar inhibitor of imidazoleglycerol phosphate dehydratase, an application rate in the range of 1 kg ha^−1^ is required for a significant herbicidal effect.^25^ In contrast, the most widely used herbicide in the world, glyphosate, also requires application rates in the range of 1–2 kg ha^−1^, although IC_50_‐values in the single and double‐digit micromolar range were reported for the target enzyme EPSP synthase from several higher plants.^26^ Compared to aspterric acid, an inhibitor of the recently discovered novel herbicide target dihydroxy‐acid dehydratase, phosphonothrixin (**1**) appears to be the stronger herbicide. Although the IC_50_‐value of aspterric acid for the *Arabidopsis* enzyme is 0.5 μM, a significant herbicidal effect on plants growing in soil was only achieved after multiple applications of aspterric acid.^27^ Taking all the data into account, we are convinced that the inhibition of DHBPS is the cause of the herbicidal effect of phosphonothrixin (**1**).

## 4 CONCLUSION

Phosphonothrixin (**1**) specifically increased the thermal stability of the riboflavin (vitamin B2) biosynthetic enzyme DHBPS and inhibited the enzyme competitively with its substrate ribulose 5‐phosphate. Binding of phosphonothrixin (**1**) to DHBPS was further confirmed by the elucidation of the X‐ray crystal structure of a DHBPS‐phosphonothrixin complex. Furthermore, the bleaching phenotype of phosphonothrixin (**1**) treated plants is a phenocopy of *Arabidopsis* antisense plants, in which a strong down‐regulation of the expression of the RIBA1 gene encoding the bifunctional enzyme DHBPS ‐ GTP cyclohydrolase II, correlated with a bleaching phenotype.^28^, ^29^ All in all, our results suggest that DHBPS inhibition is the primary mode of action of phosphonothrixin (**1**), which thereby is the first herbicide inhibiting an enzyme involved in vitamin biosynthesis.

## Supporting information


**Figure S1.** Amino acid sequence of the expressed *Arabidopsis thaliana* DHBPS protein.
**Figure S2.** Amino acid sequence of the expressed *Zymoseptoria tritici* DHBPS protein.
**Figure S3.** Thermal stability shift analysis of 6.5 μM *At*DHBPS in the presence 10 μM each of phosphonothrixin (**1**) and 16 different herbicidal compounds. The shift in melting point temperature relative to untreated *At*DHBPS (deltaT_m_) in °C is plotted against the test compounds.
**Figure S4.** Thermal stability shift analysis of 6.5 μM *At*DHBPS in the presence of different concentrations of phosphonothrixin (**1**). A K_d_‐value of 31.1 ± 2.8 μM and a deltaT_m(max)_ of 22.7 ± 0.6 °C was determined from a plot of deltaT_m_ *vs*. phosphonothrixin (**1**) concentration. The T_m_ of *At*DHBPS was determined as 42.3 ± 0.3 °C.
**Figure S5.** Kinetic characterization of the interaction of *At*DHBPS with its substrate D‐ribulose‐5‐phosphate. A K_m_‐value of 0.15 ± 0.02 mM for D‐ribulose‐5‐phosphate was determined from a plot of reaction velocity *vs*. substrate concentration.
**Figure S6.** Kinetic characterization of the interaction of *At*DHBPS with phosphonothrixin (**1**). (A) Double‐reciprocal plot of reaction velocity *vs*. D‐ribulose‐5‐phosphate concentration at different phosphonothrixin (**1**) concentrations. (B) A K_i_‐value for phosphonothrixin (**1**) of 16.6 ± 5.0 μM was determined from a replot of the slopes obtained from the double‐reciprocal plots *vs*. phosphonothrixin (**1**) concentration.
**Figure. S7.** Electron density map around phosphonothrixin (**1**) contoured at 1.5 σ.
**Figure S8.** Superposition of the phosphonothrixin (**1**)‐*Zt*DHBPS structure (gold) with the AlphaFold2 model of *At*DHBPS (cyan). Residues are numbered according to the RIB3 gene (NCBI accession number SMR48963) of Zymoseptoria *tritici* (black) and the RIBA1 gene (UniProt ID: P47924) of *Arabidopsis thaliana* (green).
**Supplemental Table S1.** X‐ray data and structure refinement statistics.

## Data Availability

The data that supports the findings of this study are available in the supplementary material of this article.
