# Supplementary material for: The herbicidal natural product phosphonothrixin is an inhibitor of the riboflavin biosynthetic enzyme L‐3,4‐dihydroxy‐2‐butanone‐4‐phosphate synthase
Source: Pest Manag Sci. 2025 Mar 24;81(8):4328–35. doi: 10.1002/ps.8791 (PMC12268802; doi:10.1002/ps.8791)
Supplement: Supplementary file 1 — Figure S1. Amino acid sequence of the expressed Arabidopsis thaliana DHBPS protein. Figure S2. Amino acid sequence of the expressed Zymoseptoria tritici DHBPS protein. Figure S3. Thermal stability shift analysis of 6.5 μM AtDHBPS in the presence 10 μM each of phosphonothrixin (1) and 16 different herbicidal compounds. The shift in melting point temperature relative to untreated AtDHBPS (deltaTm) in °C is plotted against the test compounds. Figure S4. Thermal stability shift analysis of 6.5 μM AtDHBPS in the presence of different concentrations of phosphonothrixin (1). A Kd‐value of 31.1 ± 2.8 μM and a deltaTm(max) of 22.7 ± 0.6 °C was determined from a plot of deltaTm vs. phosphonothrixin (1) concentration. The Tm of AtDHBPS was determined as 42.3 ± 0.3 °C. Figure S5. Kinetic characterization of the interaction of AtDHBPS with its substrate D‐ribulose‐5‐phosphate. A Km‐value of 0.15 ± 0.02 mM for D‐ribulose‐5‐phosphate was determined from a plot of reaction velocity vs. substrate concentration. Figure S6. Kinetic characterization of the interaction of AtDHBPS with phosphonothrixin (1). (A) Double‐reciprocal plot of reaction velocity vs. D‐ribulose‐5‐phosphate concentration at different phosphonothrixin (1) concentrations. (B) A Ki‐value for phosphonothrixin (1) of 16.6 ± 5.0 μM was determined from a replot of the slopes obtained from the double‐reciprocal plots vs. phosphonothrixin (1) concentration. Figure. S7. Electron density map around phosphonothrixin (1) contoured at 1.5 σ. Figure S8. Superposition of the phosphonothrixin (1)‐ZtDHBPS structure (gold) with the AlphaFold2 model of AtDHBPS (cyan). Residues are numbered according to the RIB3 gene (NCBI accession number SMR48963) of Zymoseptoria tritici (black) and the RIBA1 gene (UniProt ID: P47924) of Arabidopsis thaliana (green). Supplemental Table S1. X‐ray data and structure refinement statistics. [file PS-81-4328-s001.pdf]

Supplementary Information

MFVAEDDDFELDLPTPGFSSIPEAIEDIRQGKLVVVVDDEDRENEGDLVMAAQLATPEAMAFIVRHGTGIVCVSM
KEDDLERLHLPLMVNQKENEEKLSTAFTVTVDAKHGTTTGVSARDRATTILSLASRDSKPEDFNRPGHIFPLKYR
EGGVLKRAGHTEASVDLTVLAGLDPVGVLCEIVDDDGSMARLPKLREFAAENNLKVVSIADLIRYRRKRDKLVER
ASKGGRADPAFLYKVVINSKLEGKPIPNPLLGLDSTRTGHHHHHH

Figure S1. Amino acid sequence of the expressed *Arabidopsis thaliana* DHBPS protein.

MHHHHHHENLYFQGMASVNGDVSFDSIPDAVEAFAKGEFVLVMDSTNRENEGDLIIAAEDFTPAKAAFMIRHSSG
YLCAPITSALAARLELPQMVTHNSDPNRTAYTITIDAADGVTTGISAQDRSLTCRRLADPKVQKETFRRPGHIVP
LQAREGGVRVRGGHTEAGVDLCLLAGKQPVSVIAEMVQDGEEVEGKAELAGDFGMMRRDGCLAFARRYGLKVITI
EDLIRHLEANDGKKA

Figure S2. Amino acid sequence of the expressed *Zymoseptoria tritici* DHBPS protein.

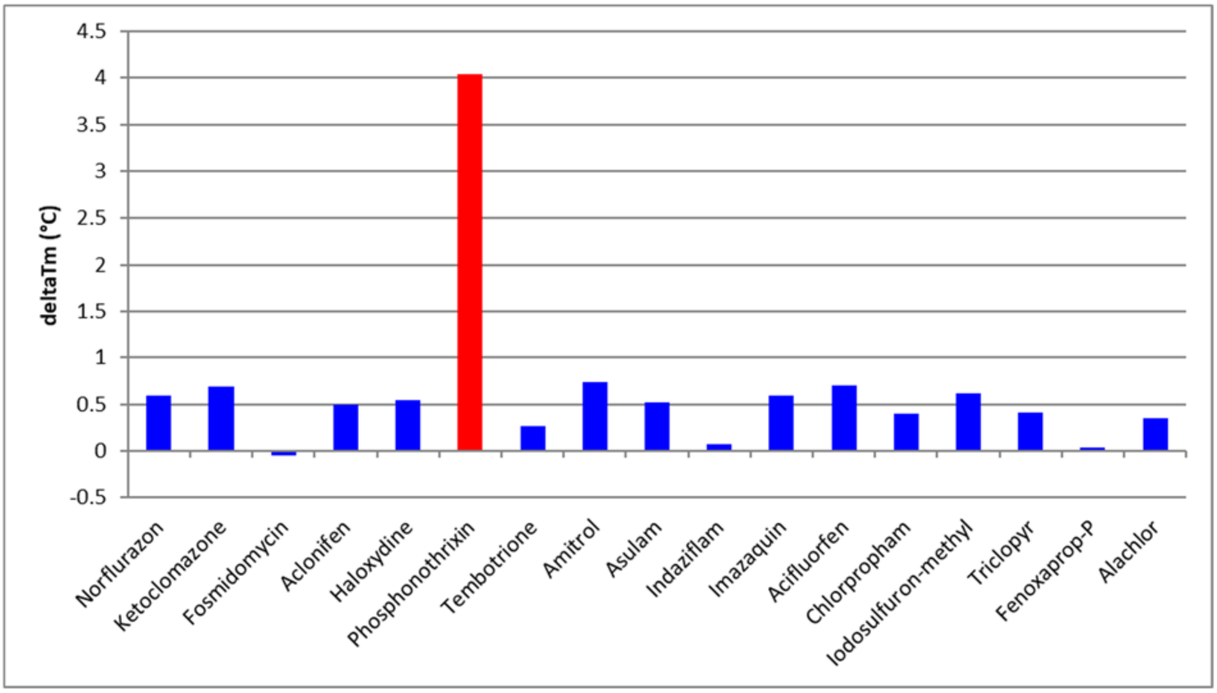

Figure S3. Thermal stability shift analysis of 6.5 µM *At*DHBPS in the presence 10 µM each of phosphonothrixin (**1**) and 16 different herbicidal compounds. The shift in melting point temperature relative to untreated *At*DHBPS ($deltaT_m$) in °C is plotted against the test compounds.

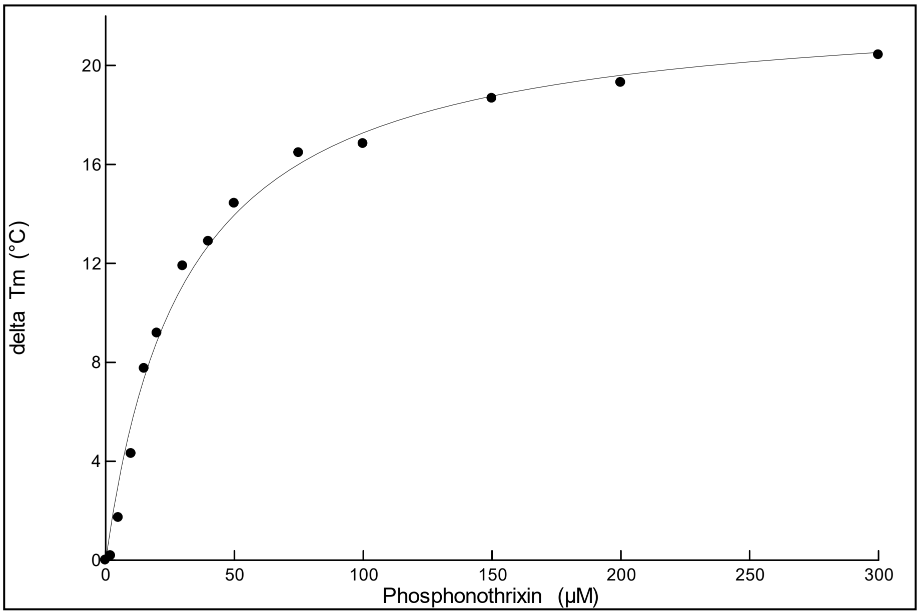

Figure S4. Thermal stability shift analysis of 6.5 µM *At*DHBPS in the presence of different concentrations of phosphonothrixin (**1**). A K_d-value of 31.1 ± 2.8 µM and a delta$T_{m(max)}$ of 22.7 ± 0.6°C was determined from a plot of deltaT_m *vs*. phosphonothrixin (**1**) concentration. The T_m of *At*DHBPS was determined as 42.3 ± 0.3°C.

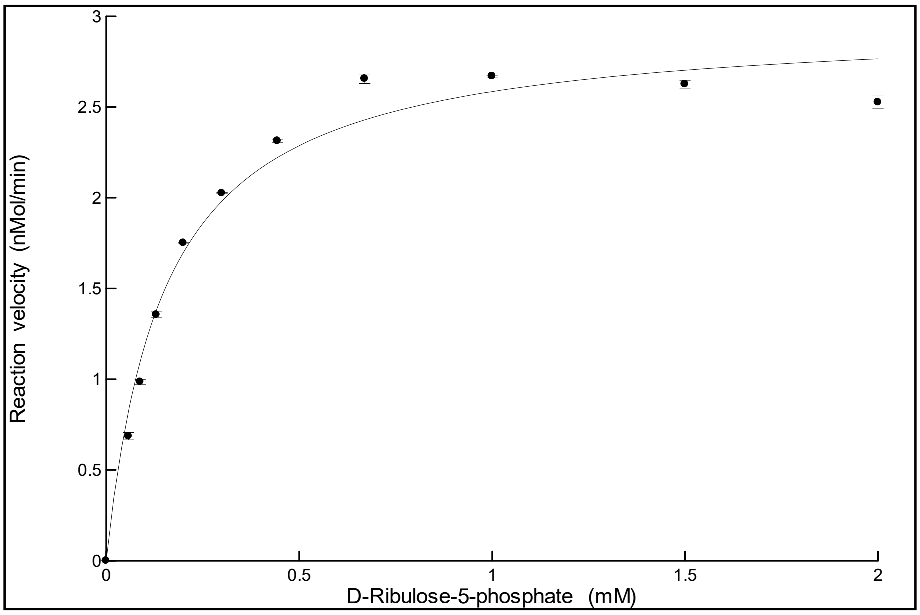

Figure S5. Kinetic characterization of the interaction of *At*DHBPS with its substrate D-ribulose-5-phosphate. A K_m-value of 0.15 ± 0.02 mM for D-ribulose-5-phosphate was determined from a plot of reaction velocity *vs*. substrate concentration.

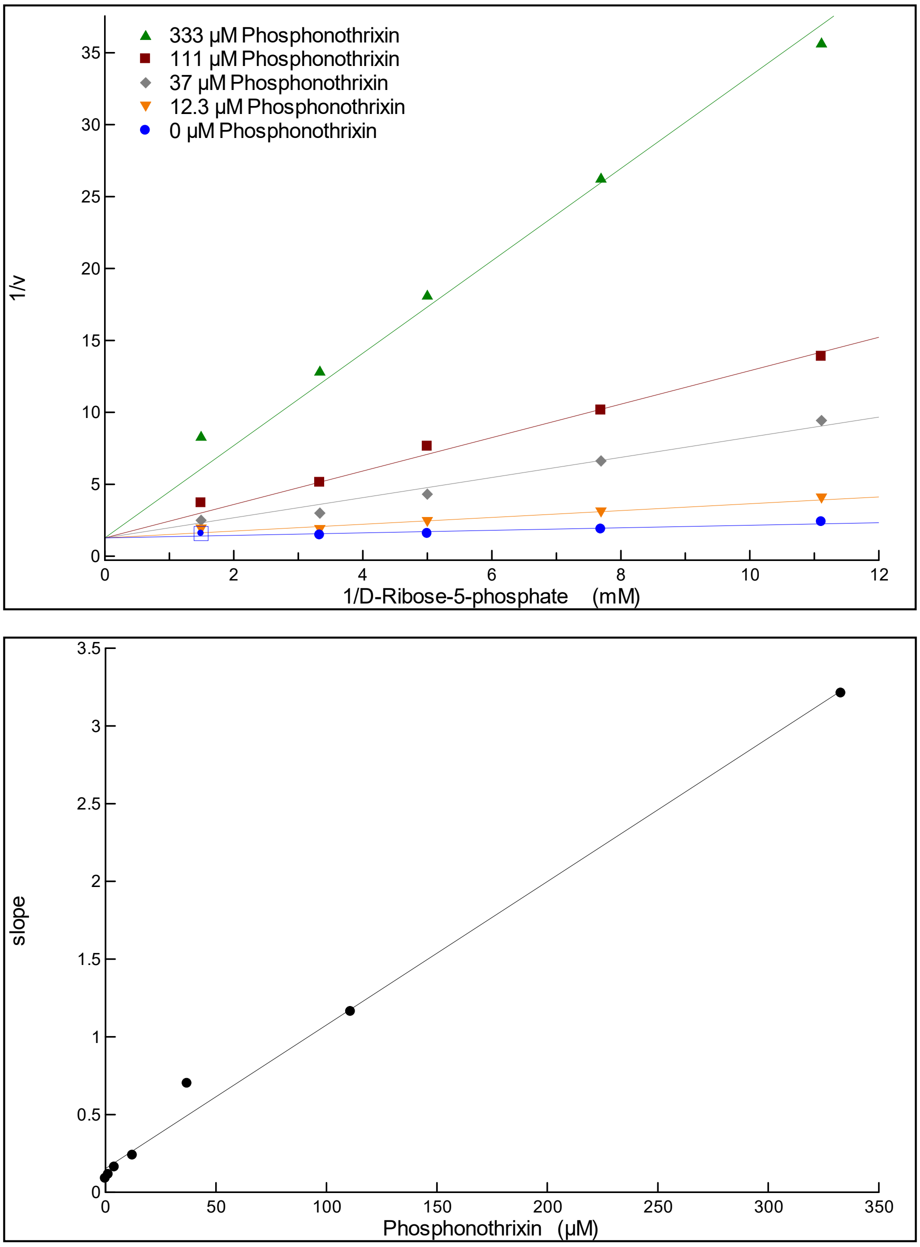

Figure S6. Kinetic characterization of the interaction of *At*DHBPS with phosphonothrixin (**1**). (A) Double-reciprocal plot of reaction velocity *vs.* D-ribulose-5-phosphate concentration at different phosphonothrixin (**1**) concentrations. (B) A K_i-value for phosphonothrixin (**1**) of 16.6 ± 5.0 µM was determined from a replot of the slopes obtained from the double-reciprocal plots *vs.* phosphonothrixin (**1**) concentration.

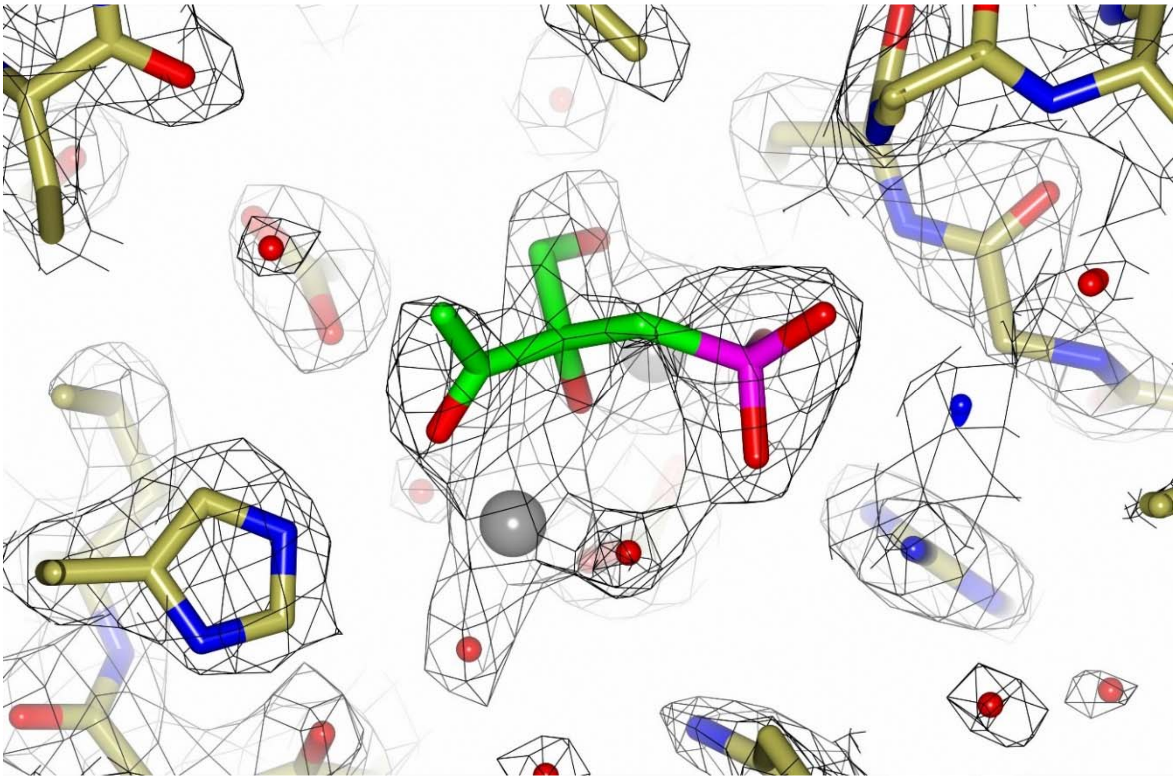

Figure S7. Electron density map around phosphonothrixin (**1**) contoured at 1.5 σ.

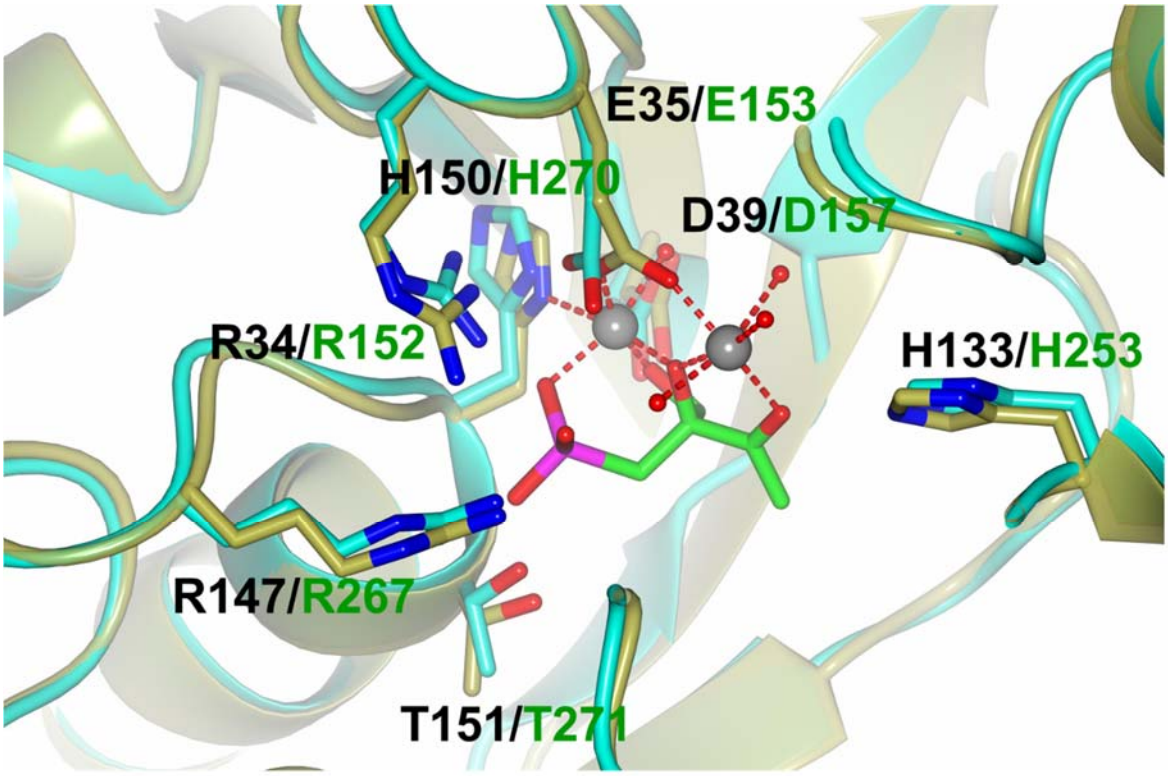

Figure S8. Superposition of the phosphonothrixin (**1**)-*Zt*DHBPS structure (gold) with the AlphaFold2 model of *At*DHBPS (cyan). Residues are numbered according to the RIB3 gene (NCBI accession number SMR48963) of Zymoseptoria *tritici* (black) and the RIBA1 gene (UniProt ID: P47924) of *Arabidopsis thaliana* (green).

Space group	P1
Cell dimensions<br>a, b, c (Å)<br>α, β, γ (°)	 42.27, 59.62, 75.45<br>110.53, 99.24, 89.94
Resolution (Å)	20-2.2 (2.32-2.20)
No. of observation	65 881 (9 679)
No. of unique reflections	37 031 (5 3062)
R_{merge} (%)	7.3 (27.8)
I/σ(I)	7.3 (2.6)
Completeness (%)	92.1 (91.3)
R_{cryst} (%)	22.6 (27.0)
R_{free} (%)	27.8 (32.6)

Values in parentheses denote the highest resolution shell

Supplemental Table 1. X-ray data and structure refinement statistics.
